# Uncovering Prognosis-Related Genes and Pathways by Multi-Omics Analysis in Lung Cancer

**DOI:** 10.3390/biom10040524

**Published:** 2020-03-30

**Authors:** Ken Asada, Kazuma Kobayashi, Samuel Joutard, Masashi Tubaki, Satoshi Takahashi, Ken Takasawa, Masaaki Komatsu, Syuzo Kaneko, Jun Sese, Ryuji Hamamoto

**Affiliations:** 1 Cancer Translational Research Team, RIKEN Center for Advanced Intelligence Project, 1-4-1 Nihonbashi, Chuo-ku, Tokyo 103-0027, Japan; ken.asada@riken.jp (K.A.); kazumakob@ncc.go.jp (K.K.); samuel.joutard@kcl.ac.uk (S.J.); sing.monotonyflower@gmail.com (S.T.); ktakazaw@ncc.go.jp (K.T.); maskomat@ncc.go.jp (M.K.); 2 Division of Molecular Modification and Cancer Biology, National Cancer Center Research Institute, 5-1-1 Tsukiji, Chuo-ku Tokyo 104-0045, Japan; sykaneko@ncc.go.jp (S.K.); sesejun@humanome.jp (J.S.); 3 National Institute of Advanced Industrial Science and Technology, Artificial Intelligence Research Center, 2-3-26, Aomi, Koto-ku, Tokyo 135-0064, Japan; tsubaki.masashi@aist.go.jp; 4 Humanome Lab, 2-4-10, Tsukiji, Chuo-ku, Tokyo 104-0045, Japan

**Keywords:** multi-omics analysis, lung cancer, survival-associated genes

## Abstract

Lung cancer is one of the leading causes of death worldwide. Therefore, understanding the factors linked to patient survival is essential. Recently, multi-omics analysis has emerged, allowing for patient groups to be classified according to prognosis and at a more individual level, to support the use of precision medicine. Here, we combined RNA expression and miRNA expression with clinical information, to conduct a multi-omics analysis, using publicly available datasets (the cancer genome atlas (TCGA) focusing on lung adenocarcinoma (LUAD)). We were able to successfully subclass patients according to survival. The classifiers we developed, using inferred labels obtained from patient subtypes showed that a support vector machine (SVM), gave the best classification results, with an accuracy of 0.82 with the test dataset. Using these subtypes, we ranked genes based on RNA expression levels. The top 25 genes were investigated, to elucidate the mechanisms that underlie patient prognosis. Bioinformatics analyses showed that the expression levels of six out of 25 genes (*ERO1B*, *DPY19L1*, *NCAM1*, *RET*, *MARCH1*, and *SLC7A8*) were associated with LUAD patient survival (*p* < 0.05), and pathway analyses indicated that major cancer signaling was altered in the subtypes.

## 1. Introduction

Lung cancer is one of the leading causes of death worldwide, mostly due to a late diagnosis. In fact, an estimated nearly 136,000 patients are expected to die from lung cancer in 2020 in the United States [1]. Even though it only contains 9% of the world’s population, Europe accounts for 25% of the global cancer burden, with an estimated 3.9 million new cancer cases and 1.9 million expected cancer deaths in 2018 [2]. Within these cases, the most common cause of cancer death was lung cancer, and 280,000 are expected to die from lung cancer in 2019 [3]. In Asia, and especially in Japan, the number of new cases of lung cancer in 2018 was 118,971 (13.5%), which is the worst number of cases among all cancers. The same was true for the risk of death; 81,820 (20.0%), as indicated by the statistics summarized by the World Health Organization (WHO) [4].

Lung cancer can be classified into two major types: small-cell lung cancer (SCLC), which accounts for approximately 15% of cases and non-small-cell lung cancer (NSCLC), which accounts for approximately 85%. Therefore, NSCLC involves the majority of the lung cancer population, and adenocarcinoma is the most common type of NSCLC. Multiple mutations have been reported to occur in NSCLC, but needless to say, the spectrum of mutations is different between different subtypes [5,6]. Thus, knowing the clinical, pathological, and molecular biological outcomes in diverse aspects is quite important to achieve an improvement in the quality of life of cancer patients.

Recently, in the medical field, deep-learning-driven classification of cancer showed a great success [7]. After that, many images-based machine-learning and deep-learning studies demonstrated their use for cancer prediction, prognosis, or even to assess treatment response in lung cancer [8,9,10]. However, single-level omics data have limitations, particularly because cancer is a heterogeneous disease, so relying on results obtained from single-level omics data may be risky and misleading; thus, it could affect the understanding of cancer as a whole and possibly negatively affect patients.

One of the proposed approaches to overcome this problem is a multi-omics analysis, an approach that has rapidly emerged in disease-related biology. A new cancer subtyping method, with the integration of multi-omics data, has already been used to reveal molecular subtypes of cancer with TCGA dataset. Multi-omics analysis, using integrated TCGA data of RNA expression, DNA methylation, point mutations, and copy number variation, demonstrated a prediction capability for poor patient outcomes [11]. Multi-omics analysis with a TCGA hepatocellular carcinoma (LIHC) dataset was also performed, using a deep-learning-based and machine-learning-based pipeline to predict patient survival, using RNA expression, DNA methylation, and miRNA expression [12]. The authors implemented an autoencoder to reduce the dimension of multi-omics features as an unsupervised approach, and then, the reduced features were further analyzed via the Cox proportional hazards (Cox-PH) model, to select survival-associated features. A similar approach was applied by using gene expression and copy number variants to classify poor or good subtypes in neuroblastoma [13].

Here, to develop a classifier for the prediction of lung-cancer-patient prognosis and to investigate a patient risk-dependent analysis, we applied a deep-learning- and machine-learning-based pipeline for multi-omics analysis of lung cancer data. We chose data of RNA expression and miRNA expression as input data, so that the result we received could be interpretable, since RNA expression is regulated by miRNA by functional duplexes. Firstly, we developed an SVM that was able to distinguish prognosis-related subtypes from the TCGA LUAD. Secondly, we performed a risk-dependent pathway analysis that can give us relevant information and knowledge about potential mechanisms related to the different subtypes. Lastly, using differentially expressing RNAs in the subtypes, we found novel genes that are associated with patient survival, and we demonstrated that newly identified genes were associated with prognosis.

## 2. Materials and Methods

### 2.1. TCGA Set

We downloaded multi-omics LUAD data from the Genomic Data Commons (GDC) TCGA data portal (https://portal.gdc.cancer.gov), using TCGA Assembler 2 (https://github.com/compgenome365/TCGA-Assembler-2; [14] with R package (R version 3.5.1). A total of 384 patients with RNA sequencing data (RNA-seq; normalized data) and miRNA sequencing data (miRNA-seq; defined using human reference genome 19 and miRBase version 20 (http://www.mirbase.org/)) were assembled into one multi-omics dataset, in the last step of the procedure. Patients’ clinical data were manually downloaded from the GDC data portal, and a total of 364 patients were available for the next analysis step. Data were preprocessed by following previous reports to deal with zero values [12]. In the last step, zero values were removed and RNA-seq data, and miRNA-seq data were standardized against patients, followed by reassembling, to make a multi-omics dataset before being fed into an autoencoder.

### 2.2. Clustering to Obtain Inferred Labels from LUAD Multi-Omics Dataset

We basically followed a pipeline and the previously published autoencoder hyper parameter settings [11]. As previously described, we implemented the autoencoder with three hidden layers (500, 100, and 500 nodes) with Keras (https://keras.io; version 2.2.4). For the hyper parameter settings, L1 and L2 regulation weights were set at 0.001 and 0.0001, respectively. Learning rate was set at 0.01, with a decay of 1e-6, and epochs were set at 150, with a dropout rate of 0.5. Stochastic gradient decent (SGD) was used as an optimizer. A bottleneck feature space of dimension 100 for each patient was extracted for further analysis.

To obtain clinically associated features from the bottleneck feature space of dimension 100, we built a univariate Cox-PH model, using a survival package in R. A log-rank *p*-value of less than 0.05 was considered as significant to select the clinical associated features.

To cluster the survival-associated features and to obtain the inferred labels, we first performed the elbow method [15], to determine the optimal clustering number in a range from one to ten. Based on the result depicted by the elbow method, we performed further analysis, to obtain the optimal number of clusters, using the Silhouette index [16] and Calinski–Harabasz criterion [17]. In the last step, based on the above results, we performed a K-means clustering, using the K, and previously determined and visualized the result with a t-Distributed Stochastic Neighbor Embedding (t-SNE) [18]. We used the scikit-learn library to perform the aforementioned clustering, and the obtained inferred labels were used to draw a Kaplan–Meier plot and then develop the classifiers described in Section 2.3 and Section 2.4.

### 2.3. Kaplan–Meier Analysis

Inferred labels obtained at clustering were used for the Kaplan–Meier analysis, to evaluate the prognosis significance of LUAD patients. Survival analysis was performed by using the R survival package, and the survival curve was drawn by using the R survminer package.

### 2.4. ANOVA Feature Ranking of miRNA and RNA Expression to Develop SVM Classifier and LUAD Prognosis-Dependent Classifiers

The multi-omics data used to draw a Kaplan–Meier plot were split into 60% for a training dataset and 40% for a test dataset. Analysis of Variance (ANOVA) method was applied to 60% training dataset to rank miRNA and RNA contributing to the subtypes. ANOVA method with the inferred labels was conducted by using the R limma package [19].

Ranked miRNAs from 5 to 20 and ranked RNAs from 5 to 30 were systematically used to develop SVM. A fixed number of miRNA and RNA were then applied to develop another three classifiers (k-nearest neighbors (KNN), Random Forest (RF), and Logistic Regression (LR)), to compare the accuracy with SVM.

### 2.5. Clinical Characterization

Two distinct populations clustered by K-means algorism were estimated, using their prognosis with their clinical information by Kaplan–Meier analysis. Clinical data used were obtained from previous reports [20], LUAD data were extracted from TCGA-CDR-Supplementary Table S1, and smoking history indicator was downloaded from the GDC data portal website [21] by selecting the “bcr biotab” option on the “Data Format” list, under the “Files” tab. On the “Cases” tab, we selected the Exposures Environmental Tobacco Smoke Exposure with the project TCGA-LUAD (file name; nationwidechildren.org_clinical_patient_luad.txt).

### 2.6. Somatic Mutation Analysis (SNPs and Small Indels)

LUAD somatic mutation data were downloaded from University of California, Santa Cruz (UCSC) Xena server (https://xenabrowser.net/datapages/) and analyzed for mutations occurring in each patient.

### 2.7. Copy Number Analysis

Log2 transformed LUAD copy number dataset was downloaded from UCSC Xena server (https://xenabrowser.net/datapages/). Copy number variant was analyzed with the platform of Affymetrix SNP 6.0 platform and assembled by hg38.

### 2.8. Pathway Analysis Enrichment in the Poor Prognosis Subtype

Gene set enrichment analysis (GSEA), Kyoto Encyclopedia of Genes and Genomes (KEGG) analysis, and Gene Ontology (GO) analysis were performed by using DESeq2, fgsea, and tidyquant packages in R, to analyze enriched pathways in the poor survival subtype.

### 2.9. Identification of the Novel Genes Associated with LUAD Patient Survival

Expression analysis was performed between the two subtypes, using the R DESeq2 package. Then, the top 25 statistically significant RNAs between the subtypes obtained from the analysis were used to analyze whether one was associated with patient survival. To draw the Kaplan–Meier plot and to obtain *p*-values from each analysis, OncoLnc web server (http://www.oncolnc.org) was used [22]. Then, 25% from the high-expression subgroup (*n* = 123) and 25% from the low-expression subgroup (*n* = 123) were used to compute prognosis. The remaining 50%, forming the intermediate-expression subgroup was excluded from the analysis.

## 3. Results

### 3.1. Subtypes Obtained from Unsupervised Approach

**Figure 1:**
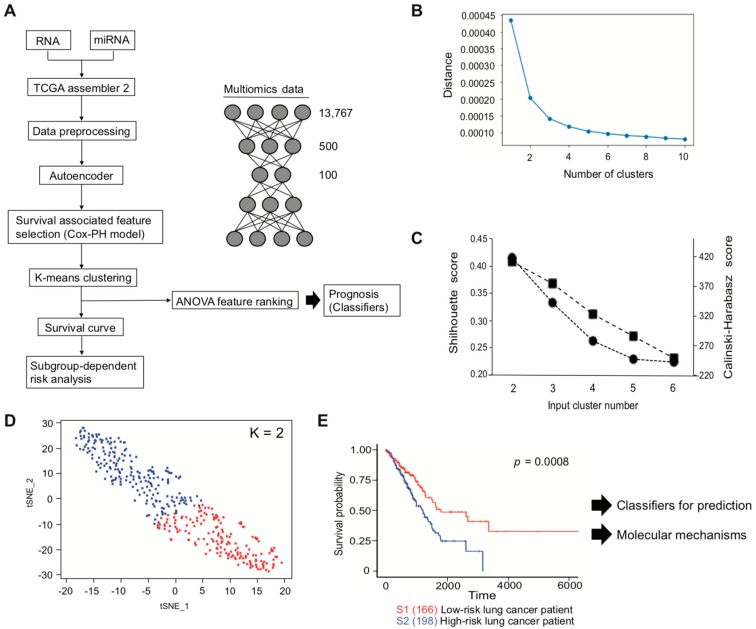
Overall workflow for classification of lung-cancer subtypes. (**A**) Multi-omics analysis pipeline. (**B**) Clustering result of elbow method. (**C**) Clustering results of the Silhouette index and Calinski–Harabasz criterion. (**D**) Clustering result of K-means clustering. Red dot represents S1, and blue dot represents S2 subtype in Figure 1E. (**E**) Kaplan–Meier plot using patient labels obtained from Figure 1D.

We used TCGA LUAD data for multi-omics analysis, to identify prognosis-related genes. Multi-omics data were generated by TCGA assembler 2, and the data were preprocessed before conducting the omics analysis described in the Materials and Methods section. A total of 13,767 features from RNA-seq and miRNA-seq data were used as an input, which were then encoded to bottleneck feature space of dimension 100 through the autoencoder (Figure 1A). To select the clinically associated features from the bottleneck features, univariate Cox-PH model was performed. In total, 33 out of 100 features showed a statistical significance by log-rank test (*p* < 0.05, Supplementary Table S1). Therefore, the 33 features were further interrogated, to determine whether they could be subcategorized depending on survival outcome. We first roughly estimated the number of clusters through the elbow method (Figure 1B) and then refined the result with more precise analyses, using the Silhouette index and Calinski–Harabasz criterion (Figure 1C; black circle (Silhouette index) and black square (Calinski–Harabasz criterion)). Both of them indicated cluster number two as an optimal number for clustering. Thus, K-means clustering was conducted with K = 2, which showed a reasonable clustering result, using t-SNE for visualization (Figure 1D). The inferred labels obtained from K-means clustering were applied to estimate patient survival, and patients were successfully sub-classed into either a poor (high-risk) or a good (low-risk) survival subtype (Figure 1E).

### 3.2. Performance of Four Classifiers Using Inferred Labels

To predict lung-cancer-patient survival, we developed several supervised classifiers for which inputs were obtained from the unsupervised autoencoder. We first considered developing an SVM model because of the previously reported prediction success using multi-omics data from TCGA LIHC [12] and the neuroblastoma project combined from Therapeutically Applicable Research to Generate Effective Treatment (TARGET) with Sequencing Quality Control [13].

For a rigorous evaluation, the multi-omics data we used to draw the Kaplan–Meier plot in Figure 1E were split into a training dataset and a test dataset. With the inferred labels, an ANOVA method was applied to the training dataset, to rank the miRNA and RNA that contribute to the subtypes (Supplementary Table S2, ranked top 20 miRNAs and top 30 RNAs). As we expected and initially speculated on obtaining the interpretable results, top ranked miRNAs were matched to the sequences of top ranked genes analyzed by TargetScanHuman web server [23] (http://www.targetscan.org/vert_72/). We then systematically used high-ranking miRNAs and RNAs, to build the SVM. The developed SVM was evaluated by using the test dataset, to estimate its accuracy. The combination of the top 20 miRNA and top 25 RNA expressions gave the best prediction results and had an accuracy of 0.82 with the test dataset (Table 1). The result of confusion matrix is as shown in Table 2.

Although the combination of the feature selection by ANOVA, followed by the development of an SVM model, gave the best performance of cancer-patient-survival prediction [13]. In this case, we investigated three additional classifiers, KNN performed with either a hyperparameter of Manhattan or Euclidean distance, RF with either a hyperparameter of Entropy or Gini impurity, and LR with either L1 or L2 regression. The best test score of KNN was 0.76, 0.67 for RF and 0.75 for LR (Table 3). As we expected, these results suggested that SVM is the best classifier if we follow the multi-omics-autoencoder-clinical-associated feature selection by Cox-PH pipeline.

### 3.3. Insight into the Genes that Are Associated with Patient Prognosis

Identifying the types of biological features is of interest, and thus, we first investigated the clinical data in the different subtypes. Table 4 shows that there were more new tumor events in the high-risk group (41.9%; (83/198)), as compared with the low-risk group (28.9%; (48/166)) (Fisher test *p* = 0.01), and that female patients tended to be in the high-risk group (58.0%; (115/198) versus 50.6%; (84/166) in the low-risk group, Fisher test *p* = 0.15). On the other hand, ages at diagnosis, tumor stages, and smoking history indicator seem to be similar in percentage in the two subtypes.

Next, we aimed to examine if the subtypes have well-investigated gene mutations, and if so, whether these vary between subtypes. We decided to analyze 18 gene mutations that were found through a comprehensive molecular profiling of TCGA LUAD [6,24]. The result is summarized in Table 5. Our findings indicate that *NF1*, a tumor-suppressor gene that negatively regulates the RAS signaling pathway was more often mutated in the high-risk subtype (14.1% versus 6.0% in the low-risk subtype, Fisher test *p* = 0.01). However, other genes, such as *TP53*, which is frequently mutated in human cancers [25], or *EGFR*, *KRAS*, and *BRAF*, which are mutations that often inform patient therapy [26], were not highly mutated in the high-risk subtype, suggesting that there may be other factors that can distinguish the different subtypes.

**Figure 2:**
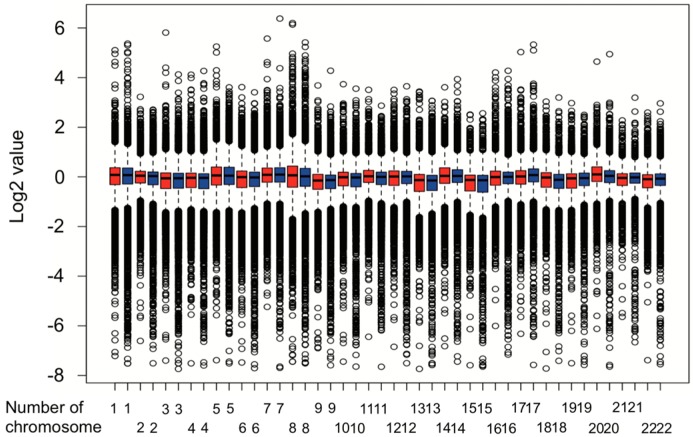
Copy number variation analysis in two subtypes. Red box represents low-risk subtype, and blue box represents high-risk subtype.

Therefore, we carried out a copy number variation analysis. Results from the copy number variation analysis were shown as Figure 2. Chromosome 2, 6, 8, 10, 11, 17, 18, 19, 20, and 22 had a different copy numbers (*p* < 0.05, Mann–Whitney U-test).

### 3.4. GSEA, KEGG Pathway Analysis, and GO Analysis

**Figure 3:**
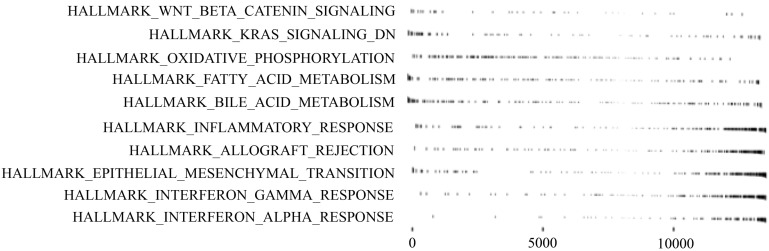
Subtype-specific signaling pathways obtained from GSEA. The left represents pathway names, and the right represents gene ranks.

Next, to explore the molecular mechanism that underlies the subtypes, differentially expressing RNA was extracted, using DESeq2 [27]. We performed a GSEA, KEGG pathway analysis, and GO analysis [28,29,30,31] to elucidate enriched pathways in the subtypes. The result of GSEA is shown in Figure 3, and the results of the KEGG and GO analyses are summarized in Table 6.

GSEA revealed that Wnt/β-catenin-signaling KRAS-signaling genes downregulated by KRAS activation that could be regulated by NF1 (Table 5), oxidative phosphorylation, and fatty acid metabolism were upregulated. Meanwhile, epithelial-mesenchymal transition (EMT) and inflammatory response, such as interferon gamma response and interferon alpha response, were downregulated in the high-risk subtype, as compared with the low-risk subtype (Figure 3). Notably, above pathways are one of the typical pathways of cancer [32,33].

KEGG pathway analysis showed that fatty acid metabolism, oxidative phosphorylation, valine, leucine, and isoleucine degradation pathways were significantly different. For the miRNA, miR-501 that activates Wnt/β-catenin signaling in gastric cancer and colorectal cancer [34,35] and tumor suppressor miR-26 that has been reported to regulate the Wnt/β-catenin signaling in prostate cancer and cholangiocarcinoma [36] were significance between subtypes. Intriguingly, miR-26 is also known to contribute TGF-β-induced EMT [37] and inflammation response [38], which could be associated with the low-risk subtype. Furthermore, miR-507 targets *KDR* (kinase insert domain receptor or VEGF receptor), and VEGF receptor is regulated by Wnt/β-catenin signaling and KRAS pathways [39]. The VEGF receptor is required in response to VEGF-dependent cell survival via EMT in colon carcinoma cell lines [40,41]. Additionally, miR-200 families are well-known miRNAs that regulate Wnt/β-catenin signaling [34] and also directly regulate EMT by targeting transcriptional repressors of *ZEB1* and *ZEB2*, which regulate *CDH1* expression [42,43]. These results indicate that the miRNAs we identified may play an important role in both high- and low-risk subtypes.

The top five GO were summarized in Table 6. Spinal cord development (GO:0021510): the spinal cord primarily conducts sensory and motor nerve impulses in the central nervous systems. The spinal cord development is co-annotated with cell–cell signaling by Wnt (GO:0198738) that is in the 6th place of 1505 co-occurring terms. Neuromuscular junction development (GO:0007528): the neuromuscular junction is the process to organize a neuromuscular junction at the cellular level. The neuromuscular junction development is co-annotated with blood vessels morphogenesis (GO:0048514) that could be associated with tumor angiogenesis in the 9th place of 1341 co-occurring terms and Wnt signalosome (GO:1990909) is also co-annotated in the 16th place of the same co-occurring therms. Cytoplasmic translation (GO:0002181); cytoplasmic translation is linked with translation (GO:0006412) and the translation is further linked with gene expression (GO:0010467), which is concordant with widely accepted knowledge that gene expression and protein synthesis are upregulated in cancer. Positive regulation of calcium ion transport (GO:0051928) is any process that activates calcium ion efflux. Cytosolic calcium ion concentration is well known to be associated with cellular functions such as gene expression, proliferation, differentiation, migration, metabolism, apoptosis, and angiogenesis [44]. Regulation of antigen receptor-mediated signaling pathway (GO:0050854) is any process that regulates signaling pathways by the cross-linking of an antigen receptor on immune cells. In particular, the relation between neuromuscular junction and cancer development has been previously demonstrated. Yes-associated protein (YAP) and β-catenin regulate synaptic differentiation and the YAP activation induced by the suppression of Hippo pathways promotes liver cancer development [45].

### 3.5. Identification of the Novel Genes Associated with LUAD Patient Survival

**Figure 4:**
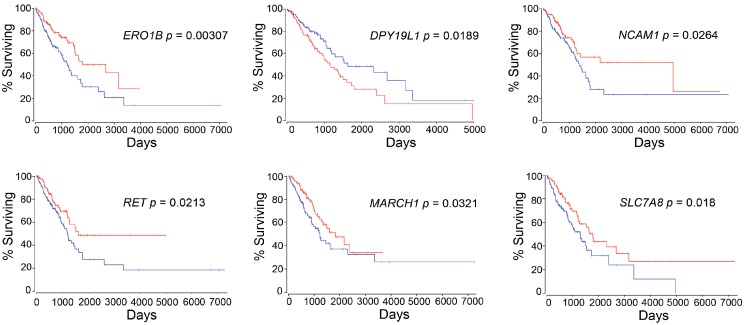
Newly identified survival-associated genes. The red line represents high-expression subtype, and the blue line represents low-expression subtype.

We focused on the top 25 differentially expressing RNAs that were extracted by using DESeq2, as shown in Table 7 and the RNA expression levels of the top 25 genes were investigated by using OncoLnc, whether they were associated with LUAD patient survival or not. Interestingly, six out of 25 genes, which are *ERO1B* (endoplasmic reticulum oxidoreductase 1 beta), *DPY19L1* (dpy-19 like C-mannosyltransferase 1), *NCAM1* (neural cell adhesion molecule 1), *RET* (ret proto-oncogene), *MARCH1* (membrane associated ring-CH-type finger 1), and *SLC7A8* (solute carrier family 7 member 8), were identified as survival-associated genes that can affect patient prognosis (Figure 4).

### 3.6. Co-Expression Analysis Reveals ERO1B, ENO3, and KCNE4 Genes Are Directed to Upregulate

**Figure 5:**
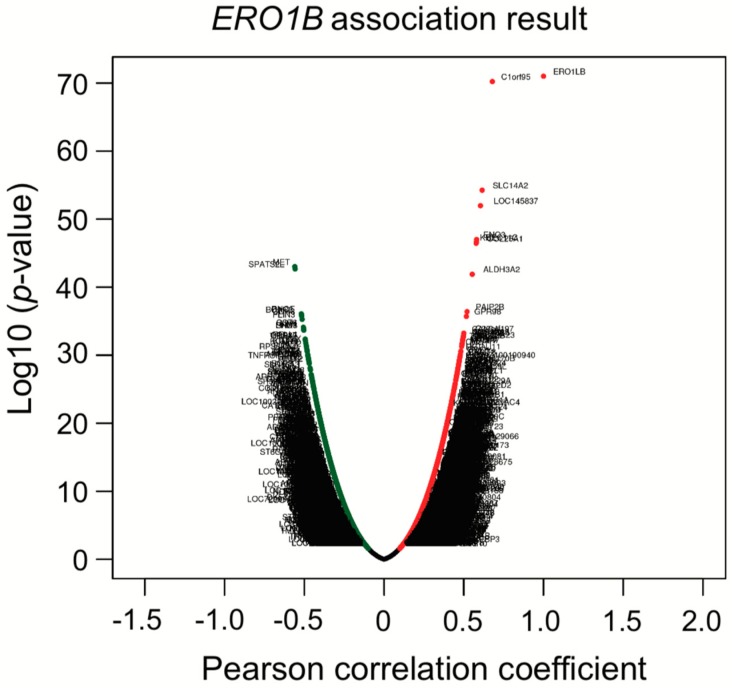
Co-expression analysis of *ERO1B*. Correlation analysis was performed with Pearson correlation test against *ERO1B* gene.

To further investigate whether the identified six genes are associated with, or potentially show, synergistic effects, a co-expression analysis was conducted with the TCGA LUAD dataset, using LinkedOmics, an interactive web-based tool [46] (http://www.linkedomics.org/login.php). Intriguingly, *ERO1B* was co-expressed with *ENO3* that is in the third place of top 25 genes, *KCNE4* that is in the seventh place, and *RET* that is in the 12th place (Figure 5; Table 7 and Table 8). Although we do not know the detailed mechanisms behind why these genes are co-expressed, epigenetic regulations or even miRNAs that can regulate multiple target genes, even with one miRNA only, may be involved. To address the abovementioned hypothesis, a miRNA target gene search was performed to find out miRNAs with a sequence that matches to the *ERO1B*, *ENO3*, *KCNE4*, and *RET* transcripts. The TargetScanHuman web server was used for the analysis, and we found that miR-6838 had a predicted to consequential pairing of *ENO3* and *KCNE4*. This suggests that the mechanism of co-expression regulation of *ENO3* and *KCNE4* may be related to the miRNA expression.

## 4. Discussion

Here, we developed a pipeline, using a TCGA LUAD dataset, with the aim of efficiently identifying genes of interest that are associated with the lung cancer patients survival. Pipeline development started with multi-omics data to implement an autoencoder, followed by clinical associated feature selection by Cox-PH. Selected features were then labeled depending on the result of K-means clustering, which is later demonstrated to be associated with patient survival. The inferred labels, or two subtypes classed by K-means clustering, were applied to plot a Kaplan–Meier survival estimation, to visualize whether the labels were associated with a poor or a good patient survival subtype and used to develop an SVM that can successfully predict patient prognosis.

During autoencoder optimization, batch size, epochs, and activation function varied. Based on our results, a batch size of 1 and epochs of 150, or even between 100 and 150, gave reasonable results, while avoiding overfitting by early stopping [47] and/or Rectified Linear Unit (ReLU) function replacing tanh function at the last layer [48] did not work well in our autoencoder. Clustering analyses applied with clinically associated features demonstrated that K = 2 was the optimal number, and this is concordant with the previous report performing with the 10 TCGA cancer dataset [49].

The multi-omics analysis with TCGA LIHC showed more *TP53* gene mutations in the high-risk subtype (Fisher test *p* = 0.042), but unfortunately, other genes such as *EGFR* were not investigated [12]. In our case, *TP53* was slightly more mutated in the high-risk subtype (0.42%), compared with the low-risk subtype (0.39%), but not significance (Fisher test *p* = 0.633). Whole-exome sequencing data of LUAD were analyzed independently in the oncogene-positive subset (*KRAS*, *EGFR*, *ERBB2*, *BRAF*, *MET*, *ALK*, *RET*, *ROS*, *HRAS*, *NRAS*, and *MAP2K1* driver mutations) and the oncogene-negative subset [24]. The authors found that *TP53* and *NF1* co-mutations were enriched in the oncogene-negative subset. Additionally, RNA profiling provided new subtypes that the proximal-inflammatory subtype (formerly squamoid) was co-mutated with *TP53* and *NF1* [6,24]. In our analysis, we found *NF1* mutations were more enriched in the high-risk subtype, suggesting that the high-risk subtype we identified might correspond to the subset that has *TP53* and *NF1* co-mutations in [24].

*ERO1B* was first reported as an endoplasmic reticulum disulfide oxidase [50]. Later, additional biological functions, such as insulin biogenesis and glucose homeostasis, were demonstrated [51]. In relation to lung cancer, *ERO1B* has been recently identified as a gene that, together with an additional three genes identified using TCGA LUAD dataset, is able to predict patient prognosis [52] and has been suggested to be a biomarker for pancreatic cancer [53,54]. *DPY19L1* was firstly identified as an unclassified gene from human brain cDNA libraries in 1998 [55]. Still, its function remains unknown, and no evidence has been reported so far on the link between *DPY19L1* and cancer prognosis. Therefore, to the best of our knowledge, this is the first report to reveal the association between *DPY19L1* expression and the prognosis in lung cancer patients. *NCAM1* or *CD56* is a member of the immunoglobulin superfamily involved in cell–cell interaction and cell–matrix interactions during the development. Additionally, it plays a fundamental role in processes such as cell migration and cell survival, in specific phenotypes of neural cells [56]. *NCAM1* may play an important role in EMT not only in intrahepatic cholangiocarcinoma but also in lung cancer via miR-200 (Table 6 and [57]). Recently, antibody-based anticancer treatment was analyzed with the expression levels of *NCAM1*. The phase 1/2 study is ongoing, since *NCAM1* is expressed on several malignancies, including SCLC [58,59,60], or could be available to predict prognosis in adult acute lymphoblastic leukemia patient [61]. *RET* was identified in 1985. *RET* is a receptor-type tyrosine kinase with multiple domains. *RET* was first discovered in papillary thyroid carcinoma, and later in sporadic tumors, neurodegenerative diseases, and Hirschsprung’s disease [62]. *RET* can be found in the rearrangement of genes generating RET fusion proteins in many cancers, including lung cancer, and thus an inhibitor was recently approved by the FDA for cancer therapy [63]. It is important to note that, not only genetic factors, but also epigenetic factors, affect *RET* expression that influences the probability of patient survival [64]. It suggests that multi-omics analysis, including epigenetic data, could improve availability of output, in terms of precision medicine or personalized medicine, as we recently reported [65]. The E3 ubiquitin ligase *MARCH1* plays an important role in immunology [66], although only a few publications have focused on *MARCH1* in the context of cancer [67,68]. Therefore, further studies in this area are required and could have the potential to contribute to the field of cancer research, and more particularly lung cancer. *SLC7A8* or *LAT-2* is an L-type amino acid transporter-2 protein that binds and regulates mechanistic target of rapamycin kinase (mTOR) activation in pancreatic cancer [69]. L-type amino acid transporters are known to be novel targets for cancer therapy [70,71]. However, as is the case for *DPY19L1* and *MARCH1*, no publications have demonstrated the link between lung cancers.

We identified six genes with expression levels that were associated with patient survival, using the autoencoder, followed by bioinformatics analysis. The practice guidelines in oncology illustrate a strategy of patient treatment based on the result of gene mutations, such as *EGFR*, *ALK*, *ROS1,* and *PD-L1* [72], but not considering RNA or miRNA expression levels. It might be of great help to estimate survival outcome and to make treatment strategy for patients if several RNA-expression levels, such as *ERO1B*, *DPY19L1*, *NCAM1*, *RET*, *MARCH1*, and/or *SLC7A8*, are also examined at the time when patients are diagnosed.

To elucidate whether six genes were only associated with LUAD patient prognosis or whether these genes were key regulators of other types of NSCLC prognosis, survival analysis against TCGA lung squamous cell carcinoma (LUSC) was performed. The *p*-values for high expression and low expression of genes of interest were from 0.106 to 0.674, suggesting that the genes we identified were LUAD-specific survival-related genes. This result gave us confidence that the multi-omics analysis we developed truly identified input-data-specific survival-associated features. In other words, if we would like to identified genes of interest that are associated with LUSC patient survival, we need to use a LUSC dataset as an input.

Co-expression analysis showed that *ERO1B*, *ENO3*, *RET*, and *KCNE4* were co-upregulated. Later, we showed that *ENO3* and *KCNE4* have a target sequence for miR-6838. The functional role of miR-6838 has been recently investigated, showing that miR-6838 regulates EMT in triple-negative breast cancer by inhibiting the Wnt pathway [73]. KEGG miRNA target analysis in Table 6 indicated that miR-26 families were enriched in the high-risk subtype. Based on the TargetScanHuman analysis, miR-26 is one of four miRNAs that was predicted to bind to the *ERO1B* transcript and suppress gene expression. As we mentioned in Section 3.4, KEGG miRNA analysis revealed that miR-501, miR-26, miR-507, miR-33, and miR-200/miR-429 were involved in lung-cancer subtypes. The miRNAs we identified have been previously reported as regulating Wnt/β-catenin signaling and/or contributing EMT signaling. Taken together, not only KEGG analysis, but also co-expression analysis, gave us insight into the molecular mechanisms that underlie patient prognosis.

A limitation of this study is the difficulty with preparing the validation dataset. The SVM model we developed uses 20 miRNA and 25 RNA expressions. Thus, we need a validation dataset that includes miRNA expression, RNA expression, and clinical information. There are datasets available that include miRNA expression (GSE63805) and RNA expression (GSE63459), together with clinical information. However, some of the miRNA expression and RNA expression for the top 20 miRNA and top 25 RNAs used to develop the SVM model were missing, and therefore we were not able to evaluate the SVM with the abovementioned publicly available dataset. This constitutes a technical limitation of the study, since it makes it difficult to assess the robustness of the developed classifier. Therefore, we decided to use the TCGA dataset again, for the validation. All data (364 patients) were randomly split into 75% and 25%, and the 25% of patient data were used for validation. Result of the accuracy score of the developed SVM model was 0.92.

The second limitation of this study is the fact the frequency of certain gene mutations can vary depending on the patients’ race. For example, EGFR mutation is more often found in Asian American patients than Caucasian or African American patients [74]. Therefore, the SVM model we developed may not be able to distinguish a high-risk subtype from a low-risk subtype if the model is applied to a different distributed dataset such as on containing an Asian population. In that case, the SVM model will need to be redeveloped.

## 5. Conclusions

Lung cancer is one of the leading causes of death worldwide. Understanding the factors that are linked with patient prognosis is essential to enhance the effectiveness of patient therapy. Recently, multi-omics analysis has emerged, allowing to classify groups of patients based on prognosis and at a more individual scale, in the context of precision medicine. Here, we only combined RNA expression, miRNA expression, and clinical information, to develop an SVM to predict patient survival in lung cancer. This enables us to significantly reduce the input omics data size, since DNA methylation data are by far bigger than other omics data; it also enables us to become interpretable.

Using bioinformatics, we established that (1) the *NF1* gene was more mutated, and (2) Wnt/β-catenin, as well as KRAS signaling pathways, can occur in the high-risk subtype. On the other hand, (3) pathways of KRAS, Wnt/β-catenin, and/or TGF-β derived EMT pathways, together with the combination of miRNA expression, could be the ones associated with low-risk subtype.

## Figures and Tables

**Table 1:** Evaluation of SVM model performance.

Number of Features (miRNA Expression + RNA Expression)	Train Score Accuracy	Test Score Accuracy
10 (5 + 5)	0.61	0.57
20 (10 + 10)	0.81	0.66
30 (15 + 15)	0.89	0.71
40 (20 + 20)	0.94	0.82
45 (20 + 25)	0.95	0.82
50 (20 + 30)	0.97	0.80

**Table 2:** Confusion matrix of SVM.

	Predicted Positive	Predicted Negative
**Positive class**	62	10
**Negative class**	17	57

**Table 3:** Evaluation of KNN, RF, and LR performance.

KNN			RF			LR		
Class	Manhattan	Euclidean	Tree	Entropy	Gini	C	L1	L2
1	0.72	0.70	1	0.54	0.54	1	0.75	0.74
2	0.71	0.68	2	0.64	0.64	5	0.72	0.75
3	0.76	0.73	3	0.64	0.66	10	0.71	0.74
4	0.73	0.74	4	0.64	0.67	50	0.70	0.71
5	0.74	0.75	5	0.66	0.67	100	0.70	0.71
6	0.71	0.72	6	0.66	0.66	500	0.70	0.69
7	0.73	0.75	7	0.67	0.65	1000	0.70	0.70
8	0.71	0.75	8	0.67	0.65			
9	0.73	0.75	9	0.67	0.65			
10	0.73	0.75	10	0.67	0.65			

**Table 4:** Clinical characterization in LUAD low-risk and high-risk subtypes.

Low-Risk (*n* = 166)		High-Risk (*n* = 198)	
Age at initial pathologic diagnosis (age)	65.5 ± 9.8	Age at initial pathologic diagnosis (age)	65.6 ± 10.2
Tumor stage *	(No.)	Tumor stage *	(No.)
Discrepancy	1	Discrepancy	2
Stage I	2	Stage I	2
Stage IA	47	Stage IA	50
Stage IB	48	Stage IB	53
Stage II	0	Stage II	1
Stage IIA	20	Stage IIA	19
Stage IIB	14	Stage IIB	29
Stage IIIA	21	Stage IIIA	31
Stage IIIB	3	Stage IIIB	4
Stage IV	10	Stage IV	7
Gender	(No.)	Gender	(No.)
Male	82	Male	79
Female	84	Female	119
Vital state	(No.)	Vital state	(No.)
Alive	116	Alive	117
Dead	50	Dead	81
Overall survival time (days)	996.0 ± 967.2	Overall survival time (days)	730.5 ± 560.0
New tumor event	(No.)	New tumor event	(No.)
Yes	48	Yes	83
No	118	No	115
Days to event	588.9 ± 539.0	Days to event	503.7 ± 444.4
Progression-free interval	(No.)	Progression-free interval	(No.)
Available	59	Available	88
Progression-free interval time (days)	836.9 ± 874.2	Progression-free interval time (days)	605.8 ± 518.3
Smoking history indicator	(No.)	Smoking history indicator	(No.)
1	22	1	29
2	39	2	45
3	46	3	48
4	54	4	69
5	0	5	4
NA or unknown	5	NA or unknown	3

* American Joint Committee on Cancer (AJCC) pathology states.

**Table 5:** Gene mutations analysis of 18 genes reported as having a statistically significant mutation in the LUAD dataset. Gene names and number of mutations (number of patients) are summarized.

Genes	Low-Risk	High-Risk
*TP53*	64 (63)	83 (80)
*KRAS*	42 (41)	40 (38)
*KEAP1*	26 (26)	27 (26)
*STK11*	20 (18)	17 (16)
*EGFR*	17 (13)	17 (13)
*NF1*	12 (10)	29 (28)
*BRAF*	12 (10)	8 (8)
*SETD2*	11 (10)	12 (10)
*RBM10*	10 (9)	11 (10)
*MGA*	10 (9)	17 (14)
*MET*	4 (4)	5 (5)
*ARID1A*	9 (8)	10 (7)
*PIK3CA*	8 (8)	9 (8)
*SMARCA4*	12 (11)	18 (18)
*RB1*	7 (6)	6 (6)
*CDKN2A*	7 (5)	6 (6)
*U2AF1*	0 (0)	0 (0)
*RIT1*	4 (3)	2 (2)

**Table 6:** Summary of KEGG pathway, miRNA, and GO analysis.

**KEGG Pathway**	***p*-Value**	**Adjusted *p*-Value**
Fatty acid metabolism	1.64 × 10^−3^	2.00 × 10^−3^
Oxidative phosphorylation	1.49 × 10^−3^	2.00 × 10^−3^
Valine, leucine and isoleucine degradation	1.61 × 10^−3^	2.00 × 10^−3^
Arachidonic acid metabolism	1.69 × 10^−3^	2.00 × 10^−3^
Pyruvate metabolism	1.64 × 10^−3^	2.00 × 10^−3^
**KEGG miRNA**	***p*-Value**	**Adjusted *p*-Value**
miR-501_AAAGGAT	1.45 × 10^−3^	0.319
miR-26a/miR-26b_TACTTGA	8.44 × 10^−3^	0.481
miR-507_GTGCAAA	8.71 × 10^−3^	0.481
miR-33_CAATGCA	6.05 × 10^−3^	0.481
miR-200b/miR-200c/miR-429_CAGTATT	2.03 × 10^−2^	0.660
**GO Analysis**	***p*-Value**	**Adjusted *p*-Value**
Spinal cord development	1.61 × 10^−3^	5.97 × 10^−2^
Neuromuscular junction development	1.64 × 10^−3^	5.97 × 10^−2^
Cytoplasmic translation	3.08 × 10^−3^	5.97 × 10^−2^
Positive regulation of calcium ion transport	2.87 × 10^−3^	5.97 × 10^−2^
Regulation of antigen receptor mediated signaling pathway	2.62 × 10^−3^	5.97 × 10^−2^

**Table 7:** Top 25 RNAs with statistical significance between the two subtypes.

Rank	Gene Name	Log2 Fold Change	*p*-Value	Adjusted *p*-Value
1	*H19*	3.45	3.01 × 10^−40^	4.08 × 10^−36^
2	*CBR1*	1.71	3.98 × 10^−27^	2.69 × 10^−23^
3	*ENO3*	1.93	1.47 × 10^−20^	6.64 × 10^−17^
4	*POLR3H*	0.89	1.22 × 10^−18^	4.12 × 10^−15^
5	*GREB1*	1.49	2.39 × 10^−18^	6.47 × 10^−15^
6	*ERO1B*	1.18	2.20 × 10^−16^	4.97 × 10^−13^
7	*KCNE4*	1.40	2.81 × 10^−15^	5.44 × 10^−12^
8	*ODC1*	1.32	5.12 × 10^−15^	8.68× 10^−12^
9	*DPY19L1*	−0.72	2.38 × 10^−14^	3.58 × 10^−11^
10	*WNT4*	1.22	5.19 × 10^−14^	7.03 × 10^−11^
11	*NCAM1*	1.37	1.48 × 10^−13^	1.82 × 10^−10^
12	*RET*	1.82	3.39 × 10^−13^	3.83 × 10^−10^
13	*ESR1*	−1.17	1.93 × 10^−12^	2.02 × 10^−9^
14	*MARCH1*	0.94	2.54 × 10^−12^	2.45 × 10^−9^
15	*SLIT1*	1.28	2.98 × 10^−12^	2.70 × 10^−9^
16	*ZNF710*	0.82	4.35 × 10^−12^	3.68 × 10^−9^
17	*GID8*	0.38	7.25 × 10^−12^	5.78 × 10^−9^
18	*CLU*	1.06	9.22 × 10^−12^	6.94 × 10^−9^
19	*AREG*	−1.37	1.07 × 10^−11^	7.66 × 10^−9^
20	*ALDH3A2*	0.72	1.15 × 10^−11^	7.80 × 10^−9^
21	*MMP11*	−1.30	1.33 × 10^−11^	8.61 × 10^−9^
22	*FAM105A*	0.98	2.33 × 10^−11^	1.43 × 10^−8^
23	*SLC7A8*	0.88	3.19 × 10^−11^	1.88 × 10^−8^
24	*BATF2*	−0.68	4.27 × 10^−11^	2.41 × 10^−8^
25	*ST3GAL3*	0.53	4.99 × 10^−11^	2.71 × 10^−8^

**Table 8:** Summary of co-expression genes.

Rank	Target Gene		Pearson Correlation	*p*-Value	FDR *
1	*C1orf95*		0.679	6.11 × 10^−71^	6.12 × 10^−67^
2	*SLC14A2*		0.615	5.63 × 10^−55^	3.75 × 10^−51^
3	*LOC145837*		0.605	1.07 × 10^−52^	5.36 × 10^−59^
4	*ENO3*	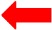	0.581	9.89 × 10^−48^	3.95 × 10^−44^
5	*SEC11C*		0.578	2.53 × 10^−47^	7.47 × 10^−44^
6	*KIT*		0.578	2.67 × 10^−47^	7.47 × 10^−44^
7	*COL25A1*		0.578	3.55 × 10^−47^	8.86 × 10^−44^
8	*MET*		−0.559	9.74 × 10^−44^	2.16 × 10^−40^
9	*SPATS2L*		−0.558	2.06 × 10^−43^	4.11 × 10^−40^
10	*ALDH3A2*		0.553	1.28 × 10^−42^	2.33 × 10^−39^
46	*RET*	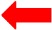	0.482	2.91 × 10^−31^	1.24 × 10^−28^
77	*KCNE4*	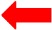	0.462	1.50 × 10^−28^	3.85 × 10^−26^

* False discovery rate (Benjamini–Hochberg procedure). Red arrows indicate ones of top 25 RNA shown in Table 7.

## Supplementary Materials

The following are available online at https://www.mdpi.com/2218-273X/10/4/524/s1. Table S1: Survival associated 33 features extracted by log-rank test. Table S2: ANOVA ranked miRNA and genes (top 20 and 25, respectively) used for SVM. Computer code: Keras code we implemented an autoencoder algorithm.
