# Supplementary material for: Uncovering Prognosis-Related Genes and Pathways by Multi-Omics Analysis in Lung Cancer
Source: Biomolecules. 2020 Mar 30;10(4):524. doi: 10.3390/biom10040524 (PMC7225957; doi:10.3390/biom10040524)
Supplement: Supplementary file 1 [file biomolecules-10-00524-s001.pdf]

Supplementary Table S1. Survival associated 33 features selected by log-rank test.

Number	Feature	Likelihood ratio test	Wald test	Log-rank test
1	Feature 1	$p = 0.009$	$p = 0.009$	$p = 0.009$
2	Feature 4	$p = 0.02$	$p = 0.02$	$p = 0.02$
3	Feature 6	$p = 0.01$	$p = 0.01$	$p = 0.01$
4	Feature 7	$p = 0.04$	$p = 0.04$	$p = 0.04$
5	Feature 15	$p = 0.008$	$p = 0.007$	$p = 0.007$
6	Feature 21	$p = 0.05$	$p = 0.05$	$p = 0.05$
7	Feature 23	$p = 0.03$	$p = 0.03$	$p = 0.03$
8	Feature 27	$p = 0.02$	$p = 0.02$	$p = 0.02$
9	Feature 28	$p = 0.02$	$p = 0.02$	$p = 0.02$
10	Feature 33	$p = 0.009$	$p = 0.009$	$p = 0.009$
11	Feature 36	$p = 0.009$	$p = 0.008$	$p = 0.008$
12	Feature 37	$p = 0.009$	$p = 0.01$	$p = 0.01$
13	Feature 38	$p = 1e-04$	$p = 1e-04$	$p = 1e-04$
14	Feature 44	$p = 0.01$	$p = 0.01$	$p = 0.01$
15	Feature 46	$p = 0.01$	$p = 0.01$	$p = 0.01$
16	Feature 48	$p = 0.02$	$p = 0.02$	$p = 0.02$
17	Feature 51	$p = 0.02$	$p = 0.02$	$p = 0.02$
18	Feature 58	$p = 0.005$	$p = 0.005$	$p = 0.005$
19	Feature 59	$p = 0.005$	$p = 0.005$	$p = 0.005$
20	Feature 61	$p = 0.02$	$p = 0.02$	$p = 0.02$
21	Feature 62	$p = 0.007$	$p = 0.007$	$p = 0.007$
22	Feature 65	$p = 0.007$	$p = 0.007$	$p = 0.007$
23	Feature 67	$p = 0.05$	$p = 0.05$	$p = 0.05$
24	Feature 71	$p = 0.003$	$p = 0.003$	$p = 0.003$
25	Feature 72	$p = 0.008$	$p = 0.007$	$p = 0.007$
26	Feature 73	$p = 0.05$	$p = 0.05$	$p = 0.05$
27	Feature 74	$p = 0.01$	$p = 0.01$	$p = 0.01$
28	Feature 79	$p = 3e-04$	$p = 3e-04$	$p = 3e-04$
29	Feature 82	$p = 0.04$	$p = 0.04$	$p = 0.04$
30	Feature 84	$p = 4e-05$	$p = 5e-05$	$p = 5e-05$
31	Feature 87	$p = 0.04$	$p = 0.04$	$p = 0.04$
32	Feature 92	$p = 0.005$	$p = 0.005$	$p = 0.005$
33	Feature 100	$p = 0.04$	$p = 0.04$	$p = 0.04$

Supplementary Table S2. ANOVA ranked miRNA and genes (top 20 and 30, respectively) used for SVM.

Number	miRNA	Gene
1	miR-21	*SFTPB*
2	miR-375	*ADAM6*
3	miR-143	*H19*
4	miR-10a	*LYZ*
5	miR-99b	*FTL*
6	miR-10b	*FN1*
7	miR-148a	*MUC5B*
8	miR-22	*SFTPA2*
9	Let-7b	*S100A9*
10	miR-30a	*CD74*
11	miR-182	*CPM*
12	miR-183	*LOC96610*
13	miR-92a-2	*IGF2*
14	miR-203a	*ACTB*
15	miR-29a	*GAPDH*
16	miR-200c	*TMSB4XP8*
17	Let-7a-2	*ALDH1A1*
18	miR-100	*SLC34A2*
19	miR-103a-1	*ERBB2*
20	miR-30d	*FTH1*
21	NA	*KRT18*
22	NA	*TPT1*
23	NA	*COL3A1*
24	NA	*COL1A1*
25	NA	*ACTG1*
26	NA	*KRT8*
27	NA	*EEF1A1*
28	NA	*B2M*
29	NA	*RPL3*
30	NA	*ENO1*

Supplementary Computer Code. Keras code we implemented an autoencoder algorithm.

```
inp = Input(shape=(13767,))

encoded = Dense(500, activation='tanh',
        kernel_regularizer=regularizers.l2(10e-3),
        activity_regularizer=regularizers.l1(10e-4))(inp)

encoded = Dropout(0.5)(encoded)
encoded = Dense(100, activation='tanh',
        kernel_regularizer=regularizers.l2(10e-3),
        activity_regularizer=regularizers.l1(10e-4))(encoded)

encoded = Dropout(0.5)(encoded)
decoded = Dense(500, activation='tanh')(encoded)
decoded = Dense(13767, activation='tanh')(decoded)

autoencoder = Model(inp, decoded)
sgd = optimizers.SGD(lr=0.01, decay=1e-6, momentum=0.9, nesterov=True)
autoencoder.compile(optimizer='sgd',
          loss='mean_squared_error',
          metrics=['accuracy'])

history = autoencoder.fit(X, X,
              epochs=150,
              batch_size=1)
```
